# Therapeutic efficacy of deslanoside in oleic acid-induced acute lung injury in rabbits: Implications for clinical drug use and pharmacological strategies

**DOI:** 10.3389/fphar.2025.1683838

**Published:** 2025-10-30

**Authors:** Rong Hu, Yi Zheng, Ning Li, Yong Pan, Xiaozhen Zhou, Lu Liu, Dizheng Liao, Yafei Liu

**Affiliations:** ^1^ Department of Ultrasound, Zhuzhou Central Hospital, Zhuzhou, Hunan, China; ^2^ Department of Cardiology, Zhuzhou Central Hospital, Zhuzhou, Hunan, China; ^3^ Department of Neonatology, Zhuzhou Central Hospital, Zhuzhou, Hunan, China

**Keywords:** deslanoside, acute lung injury, therapeutic efficacy, inflammation, drug repositioning, pharmacological strategy

## Abstract

**Background:**

Acute lung injury (ALI) remains a life-threatening condition with limited effective pharmacological options. Deslanoside, a cardiac glycoside traditionally used in heart failure, has recently attracted attention for its anti-inflammatory and tissue-protective properties.

**Objective:**

This study evaluated the therapeutic efficacy of deslanoside in an oleic acid-induced rabbit ALI model and discussed its potential clinical and pharmacological implications. Deslanoside is known to modulate Na^+^/K^+^-ATPase–related signaling and inhibit the NF-κB–mediated inflammatory cascade, which may contribute to its protective effects in ALI.

**Methods:**

Thirty healthy male New Zealand white rabbits were randomly assigned to control (saline) or experimental (deslanoside) groups following intravenous oleic acid injection to induce ALI. Modified lung ultrasound (MLUS) scores, arterial blood gas analysis, lung water content, histopathology, and serum levels of TNF-α, IL-1β, and IL-6 were assessed over 12 h.

**Results:**

Compared with controls, deslanoside treatment significantly improved PaO_2_, reduced MLUS scores, decreased lung water content, and lowered histopathological injury scores (all P < 0.05). Inflammatory cytokine levels were also markedly reduced (P < 0.05). No acute adverse drug reactions were observed.

**Conclusion:**

Deslanoside demonstrated significant protective effects in oleic acid-induced ALI, improving oxygenation, attenuating pulmonary edema, and reducing inflammation. These findings support the potential repositioning of deslanoside as an adjunctive therapy for ALI and provide experimental evidence to inform future clinical drug use strategies and pharmacological policy discussions.

## Introduction

Acute Lung Injury (ALI) and its more serious form, Acute Respiratory Distress Syndrome (ARDS), are common and dangerous diseases in clinical intensive care. It is characterized by high morbidity and mortality (Wang et al., 2024; Cai et al., 2025). These diseases are usually triggered by a variety of internal and external factors, such as severe infection, trauma, shock, inhalation injury or drug poisoning, which lead to uncontrolled inflammatory response in the lungs, and then lead to extensive damage of alveolar epithelial cells and capillary endothelial cells, resulting in a series of pathophysiological changes such as pulmonary edema, hemorrhage, hypoxemia and respiratory dysfunction. Despite the significant progress in mechanical ventilation, fluid management and anti-infective therapy in recent years, the mortality of ALI/ARDS remains high and has become a medical problem to be solved worldwide (Yin et al., 2025; Cheng et al., 2024).

As a classical experimental ALI model, oleic acid-induced acute lung injury (ALI) in rabbits has been widely used to study the pathogenesis of ALI and to evaluate new therapeutic methods and drug efficacy. This model simulates the process of lung tissue injury induced by lipid peroxidation *in vivo* by intravenous injection of oleic acid, and can reproduce the pathological characteristics of human ALI, such as pulmonary edema, inflammatory cell infiltration, alveolar collapse and gas exchange disorders, which provides a reliable experimental platform for further study of ALI (Tian et al., 2025; Guo et al., 2014). Deslanoside, a cardiac glycoside extracted from natural plants, has been traditionally used to treat cardiovascular diseases such as heart failure. In recent years, with the in-depth study of its pharmacological effects, it has been found that deslanoside not only has cardiotonic, diuretic and vasodilator effects, but also has potential therapeutic value for a variety of inflammatory diseases by regulating immune response and inhibiting the release of inflammatory mediators (Liu et al., 2021; Chen et al., 2012; Helke et al., 1978; Bakke et al., 1981). Recent studies have demonstrated that deslanoside can modulate Na^+^/K^+^-ATPase–related signaling pathways, inhibit the activation of NF-κB and MAPK cascades, and suppress the expression and release of pro-inflammatory cytokines such as TNF-α, IL-1β, and IL-6 (Fürst et al., 2017). In addition, deslanoside may reduce oxidative stress, stabilize endothelial cell function, and preserve alveolar–capillary barrier integrity, thereby alleviating inflammatory damage and pulmonary edema. These mechanisms provide a strong biological rationale for the drug-repurposing potential of deslanoside in treating ALI/ARDS (El-Seedi et al., 2022). Especially in the field of pulmonary diseases, some studies have shown that deslanoside may have a certain therapeutic effect on ALI/ARDS by reducing pulmonary inflammation, improving pulmonary circulation and promoting the absorption of pulmonary edema (Gonzales et al., 2015; Silva et al., 2023). However, its specific mechanism of action and clinical application effect need further research and verification.

The aim of this study was to systematically evaluate the therapeutic effect of deslanoside on ALI and its potential mechanism by constructing a rabbit model of acute lung injury induced by oleic acid, and to provide a new theoretical basis for the clinical treatment of ALI/ARDS.

## Materials and methods

### Ethical approval

All experimental procedures were approved by the Animal Ethics Committee of Central South University and conducted in accordance with the institutional guidelines for animal experimentation.

## Animals

Thirty healthy male New Zealand white rabbits (2.5–3.0 kg) were obtained from the Laboratory Animal Center of Central South University. The sample size was determined by power analysis with α = 0.05, β = 0.20, and an expected correlation coefficient of 0.5, which yielded a minimum required sample size of 28 subjects. Animals were housed in a standard environment (temperature 20 °C–26 °C, relative humidity 55%–65%) with natural lighting and free access to food and water. Rabbits were fasted for 6 h before the experiment but had free access to water.

### Model establishment

After a 6-h fasting period, rabbits were weighed and fixed in position. The hair over the left marginal ear vein was removed using a clipper, and the area was disinfected. A scalp needle was inserted into the marginal ear vein, and 3% pentobarbital sodium (1.25 mL/kg) was slowly injected through the needle, followed by flushing with 1 mL of 0.9% normal saline. After anesthesia, rabbits were fixed in a supine position on the rabbit board, and a warm air blower was used to maintain body temperature. Oleic acid (95% purity, analytical grade) was injected through the marginal ear vein at a dose of 0.12 mL/kg to establish the pulmonary edema model. Fifty minutes after injection, transthoracic lung ultrasound examination was performed bilaterally along the midclavicular line and anterior axillary line in longitudinal sections to observe each intercostal space. The presence of B-lines was recorded, and images were stored. The appearance of increased B-lines in any intercostal space indicated successful model establishment, if no increased B-lines were observed in any intercostal space, the model was considered unsuccessful. After successful modeling, deslanoside (0.25 mg/mL, 0.10 mL/kg) was administered intravenously through the marginal ear vein immediately following confirmation of ALI, while the control group received an equal volume of normal saline. The dosage of deslanoside was selected based on previous animal studies and pharmacodynamic data demonstrating effective systemic exposure with acceptable cardiac safety margins in rabbits and rodents (Liu et al., 2021; Chen et al., 2012). This dose was also consistent with prior research evaluating the cardiovascular and anti-inflammatory activities of cardiac glycosides.

### Blood gas analysis

The right femoral artery was cannulated under the guidance of ultrasound, and a 24 G indwelling needle was inserted and fixed with a heparin cap as the access for arterial blood gas analysis. Arterial blood 2 mL was collected from the femoral artery and the partial pressure of oxygen (PaO2) was measured immediately using a fully automated blood gas analyzer (GEM Premier 3500, Instrument Laboratories, Inc.).

### Ultrasonography

A Mindray M9 color Doppler ultrasound diagnostic instrument (Mindray, Shenzhen, China) was used, equipped with a linear array probe operating at a frequency of 6–15 MHz. The left and right sides of the anterior chest wall of the experimental rabbits were divided into upper and lower lung regions, and ultrasound scanning was performed in turn along the intercostal space to avoid rib occlusion as far as possible. The sum of ultrasound scores of the above four lung regions was MLUS. Each experimental rabbit was observed and recorded according to the MLUS standard at the above four time points. MLUS scoring criteria are shown in Table 1. A “B-line” was defined as a discrete, vertical, hyperechoic artifact that originated from the pleural line, extended to the bottom of the screen without fading, and moved synchronously with lung sliding, representing alveolar–interstitial syndrome.

**TABLE 1:** MLUS scoring criteria.

Ultrasound signs	Score of each lung area (points)
Lung parenchyma condition	
no B-lines	0
≤3 B-lines	1
>3 B-lines or part of the B lines are fused	2
All B lines are fused (white lung or waterfall lung)	3
Pulmonary consolidation or subpleural lesions	4
Pleural line	
Normal	0
Thickening (≥0.5 mm)	1
Unclear or irregular	2
Discontinuous and fragmented	3
Complications	
Am line	
No	0
Yes	4
Pleural effusion	
No	0
Yes	5

### Lung histopathological examination

The rabbits were sacrificed at the end of the above examinations 12 h after modeling, and the gross lung tissue was observed with naked eyes. The whole lung was sampled according to the four lung regions and stained with HE. Three non-overlapping visual fields were obtained from each section under the light microscope to observe whether there were pulmonary hemorrhage, capillary congestion, pulmonary interstitial inflammatory cell infiltration, alveolar wall thickening or hyaline membrane formation. The pathological score of each index was the average of the three visual fields counted in each section. Pathological scoring criteria: 0 point for no obvious injury, one point for injury ≤25% of the whole visual field area, two points for injury accounting for 25%∼50% of the whole visual field area, three points for >50%∼75%, and four points for >75%. The sum of the average values of the four indicators was the pathological score of the section.

### Water content of lung tissue

The rabbits were sacrificed 12 h after the establishment of the model, the abdomen of the rabbits was flattened upward, the chest was shaved, routine disinfection was carried out, and the right lung was removed; Gently clip the lung tissue, pay attention to clip the surrounding connective tissue, do not damage the lung tissue to avoid artificial bleeding points, rinse it in saline for three times, remove the excess connective tissue, and use absorbent paper to absorb the water on the surface of the tissue; The lung tissue was immediately weighed and then placed in a 55 °C oven for 48 H, and then weighed again to determine the dry/wet weight ratio for the assessment of pulmonary edema.

### Serum levels of TNF-α, IL-1β and IL-6 were measured by ELISA

At 12 h after injury, about 2 mL of blood was taken from the heart and centrifuged at 2000 rpm/min for 10 min at 4 °C for dilution. Serum levels of TNF-α, IL-1β and IL-6 were measured by ELISA.

### Statistical analysis

In this study, SPSS 26.0 software was used to process and analyze the relevant data. The measurement data that did not conform to the normal distribution were expressed by median and interquartile range, and the measurement data that conformed to the normal distribution were expressed by mean ± standard deviation (X ± s). The independent sample t-test was used for comparison between the two groups, and the paired t-test was used for comparison within the group. Enumeration data were expressed as rate or constituent ratio, chi-square test or Fisher’s exact test was used to compare the two groups, and Wilcoxon rank sum test was used to test the rank data. P < 0.05 was considered statistically significant.

## Results

### Comparison of PaO2 between the two groups

The partial pressure of blood oxygen in the experimental group was significantly higher than that in the control group at 4 H, 8 H and 12 H, and the differences between them were statistically significant (P < 0.05), At the 1-h time point, no significant difference in PaO_2_ was observed between the two groups (P = 0.113), which may be attributed to the short time interval after oleic acid injection, during which the pathological changes and pharmacological effects of deslanoside were not yet fully manifested. The improvement in oxygenation became evident after 4 h, corresponding to the progression of lung injury and the onset of deslanoside’s anti-inflammatory and anti-edematous effects, as shown in Table 2.

**TABLE 2:** Comparison of PaO2 between the two groups.

Point in time	Control group	Experimental group	t value	P value
1 h	86.21 ± 6.31	88.69 ± 7.02	0.896	0.113
4 h	84.33 ± 6.18	89.71 ± 7.15	2.654	0.046
8 h	82.44 ± 5.87	91.28 ± 7.62	5.372	<0.001
12 h	79.16 ± 5.25	92.59 ± 7.93	7.583	<0.001

## Comparison of MLUS scores between the two groups

**FIGURE 1:**
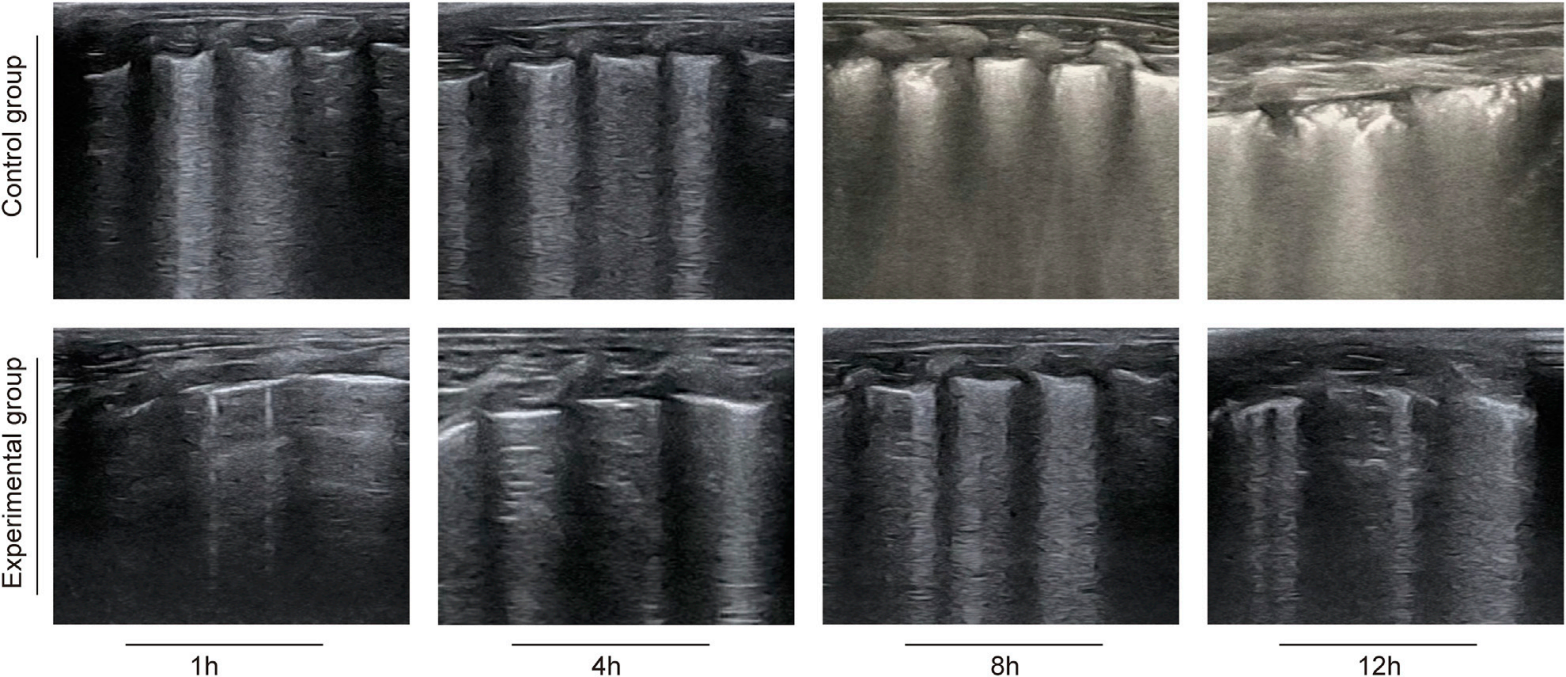
Representative lung ultrasound images in rabbits with oleic acid–induced acute lung injury.

Compared with the control group, the MLUS scores of the experimental group at 4 H, 8 H and 12 H were significantly lower, and the differences between them were statistically significant (P < 0.05), as shown in Table 3. Representative MLUS images are shown in Figure 1. In the control group, the number and density of vertical hyperechoic artifacts (B-lines) progressively increased over time, with partial fusion and “white-lung” appearance observed at 8–12 h, indicating worsening pulmonary edema and alveolar–interstitial syndrome. In contrast, in the deslanoside-treated experimental group, fewer and thinner B-lines were observed, with better pleural line continuity and less ultrasonic evidence of interstitial fluid accumulation. These qualitative imaging findings are consistent with the quantitative MLUS scores, further confirming that deslanoside effectively alleviated pulmonary edema and improved lung aeration in rabbits with ALI.

**TABLE 3:** Comparison of MLUS scores between the two groups.

Point in time	Control group	Experimental group	t value	P value
1 h	20.43 ± 6.78	14.35 ± 4.26	4.781	<0.001
4 h	25.67 ± 7.91	17.23 ± 5.81	6.196	<0.001
8 h	30.45 ± 8.26	19.97 ± 6.04	8.243	<0.001
12 h	34.23 ± 9.17	23.71 ± 6.22	10.786	<0.001

### Comparison of lung histopathological scores between the two groups

Compared with the control group, the lung histopathological score of the experimental group was significantly lower, and the difference between them was statistically significant (P < 0.05), as shown in Table 4.

**TABLE 4:** Comparison of lung histopathological scores between the two groups.

Group	Case	Lung histopathological scores
Control group	n = 15	21.69 ± 5.17
Experimental group	n = 15	11.35 ± 4.02
t value		8.961
P value		<0.001

## Comparison of lung water content (%) between the two groups

The water content of lung tissue in the experimental group was significantly lower than that in the control group, and the difference was statistically significant (P < 0.05), as shown in Table 5. The acute pulmonary edema of rabbits in the control group was more serious.

**TABLE 5:** Comparison of lung water content (%) between the two groups.

Group	Case	Lung water content (%)
Control group	n = 15	89.73 ± 4.97
Experimental group	n = 15	78.98 ± 4.65
t value		7.432
P value		<0.001

### Comparison of serum TNF-α, IL-1β and IL-6 levels between the two groups

The serum levels of TNF-α, IL-1β and IL-6 in the experimental group were significantly lower than those in the control group, and the differences between them were statistically significant (P < 0.05), as shown in Table 6.

**TABLE 6:** Comparison of serum TNF-α, IL-1β and IL-6 levels between the two groups.

Group	Case	TNF-α	IL-1β	IL-6
Control group	n = 15	198.26 ± 24.18	125.43 ± 21.69	215.43 ± 28.79
Experimental group	n = 15	113.79 ± 18.71	75.96 ± 14.38	145.12 ± 22.16
t value		24.578	17.683	21.487
P value		<0.001	<0.001	<0.001

## Discussion

Acute lung injury (ALI) is a common critical disease with high morbidity and mortality, which is a serious threat to the life and health of patients. Its pathogenesis is complex, involving uncontrolled inflammatory response, oxidative stress injury, alveolar-capillary barrier dysfunction and other links (Matthay et al., 2012; Alonso-Ojembarrena et al., 2024; Bhattacharya and Matthay, 2013). At present, the clinical treatment of acute lung injury is relatively limited, and the therapeutic effect is not ideal, so it is urgent to find new effective treatment drugs and methods (Trieu and Qadir, 2024; Przybyl et al., 2025). In this study, a rabbit model of acute lung injury induced by oleic acid was established to explore the therapeutic effect of deslanoside and its potential mechanism.

The results of this study showed that the partial pressure of blood oxygen in the experimental group was significantly higher than that in the control group at 4 H, 8 H and 12 H. As a key indicator of pulmonary gas exchange function, the increase of partial pressure of oxygen indicates that deslanoside can effectively improve oxygenation in rabbits with acute lung injury induced by oleic acid. When acute lung injury occurs, oleic acid will cause pulmonary inflammation after entering the lungs, resulting in the damage of alveolar epithelial cells and vascular endothelial cells, the increase of alveolar-capillary membrane permeability, and then affect the efficiency of gas exchange, resulting in the decrease of blood oxygen partial pressure (Zheng et al., 2024; Golding et al., 2024). Deslanoside may play a role in improving this situation through a variety of ways.On the one hand, it may reduce the damage of inflammation to lung tissue, protect the integrity of alveolar epithelial cells and vascular endothelial cells, thereby maintaining the normal structure and function of alveolar-capillary membrane and reducing gas exchange disorders. On the other hand, deslanoside may regulate the hemodynamics of the lungs, improve blood circulation in the lungs, enable oxygen to be more effectively transported to the blood, and thus increase the partial pressure of oxygen (Zhou et al., 2024; Wang et al., 2025).

Compared with the control group, the MLUS scores at 4 H, 8 H and 12 H were significantly decreased in the experimental group. Lung ultrasound score (MLUS) can directly reflect the degree of lung lesions, the lower the score, the lighter the lung lesions. The effect of deslanoside on MLUS score may be related to the reduction of pulmonary edema and improvement of pulmonary ventilation.In the process of acute lung injury, the inflammatory response will lead to increased pulmonary vascular permeability, fluid exudation into the alveoli and pulmonary interstitium, and the formation of pulmonary edema, which will seriously affect the ventilation and ventilation function of the lungs. Deslanoside may reduce the degree of pulmonary edema by inhibiting the release of inflammatory mediators and reducing the leakage of pulmonary blood vessels.At the same time, it may help to improve the compliance of the lungs, so that the lungs can ventilate better, and then show lower scores in lung ultrasound (Oulego-Erroz et al., 2024; Su et al., 2024).

The lung histopathological score of the experimental group was significantly lower than that of the control group, and the lung water content of the experimental group was significantly lower than that of the control group. These results indicate that deslanoside can significantly reduce the pathological damage of lung tissue and the degree of pulmonary edema in rabbits with acute lung injury induced by oleic acid. From the pathological point of view, oleic acid-induced acute lung injury can cause inflammatory cell infiltration, hemorrhage, edema and other pathological changes in lung tissue. Deslanoside may alleviate the pathological changes of lung tissue by inhibiting the activation and infiltration of inflammatory cells and reducing the damage of inflammatory mediators to lung tissue. At the same time, it may further reduce pulmonary edema by regulating water and salt metabolism, promoting the absorption and discharge of lung fluid, and reducing the water content of lung tissue.This alleviation of pathological damage to lung tissue and pulmonary edema helps to protect the normal structure and function of lung tissue and provides favorable conditions for the repair and recovery of the lung (Millar et al., 2022; Liu et al., 2023).

The serum levels of TNF-α, IL-1β and IL-6 in the experimental group were significantly lower than those in the control group. This indicates that deslanoside can effectively inhibit the inflammatory response in oleic acid-induced acute lung injury. Inflammation plays a key role in the development of acute lung injury. When oleic acid enters the lungs, it will activate immune cells in the lungs, such as macrophages and neutrophils, and release a large number of inflammatory factors, such as TNF-α, IL-1β and IL-6. These inflammatory factors will further promote the infiltration and activation of inflammatory cells, form an inflammatory cascade, and aggravate the damage of lung tissue. Deslanoside may reduce the synthesis and release of inflammatory cytokines by inhibiting the activation of immune cells and the conduction of inflammatory signaling pathways. It may act on intracellular signal transduction molecules to block the transmission of inflammatory signals, thereby down-regulating the expression of inflammatory factors. In addition, deslanoside may also inhibit the inflammatory response by regulating the immune function of the body and enhancing the anti-inflammatory response (Škubník et al., 2021; Evans and Seifert, 2025). In addition to its general anti-inflammatory and cardiotonic properties, deslanoside may exert its therapeutic effects in ALI by modulating Na^+^/K^+^-ATPase–associated signaling and downstream suppression of the NF-κB and NLRP3 inflammasome pathways. By inhibiting NF-κB–dependent transcription of pro-inflammatory cytokines (TNF-α, IL-1β, IL-6) and attenuating oxidative stress, deslanoside may help preserve alveolar–capillary barrier integrity, reduce endothelial permeability, and alleviate pulmonary edema. This is consistent with the mechanism proposed for other cardiac glycosides such as digoxin and ouabain, which have also been shown to inhibit inflammatory cascades and mitigate tissue injury in ALI or sepsis-related lung injury models (Škubník et al., 2021; Wang et al., 2018; Omole et al., 2025). Although our study did not perform direct molecular validation, the results collectively suggest that deslanoside may modulate the NF-κB/NLRP3 signaling axis to exert its anti-inflammatory and anti-edematous effects.

The oleic acid-induced ALI model used in this study primarily reflects lipid peroxidation–mediated endothelial and epithelial injury, reproducing key features of the early exudative phase of human ALI/ARDS, such as diffuse alveolar damage, increased alveolar–capillary permeability, and permeability edema. However, it does not fully replicate the complex immune dysregulation and multi-organ interactions observed in sepsis- or trauma-induced ARDS. Therefore, the generalizability of our findings should be interpreted with caution. In future studies, we plan to validate the therapeutic effects of deslanoside in complementary ALI/ARDS models, including LPS- or CLP-induced sepsis, ventilator-induced lung injury, and acid aspiration models, to better approximate the pathophysiological diversity of human ARDS.

To sum up, deslanoside has a significant therapeutic effect on oleic acid-induced acute lung injury in rabbits, which can effectively reduce the degree of acute lung injury by improving blood oxygen partial pressure, reducing MLUS score, alleviating pathological damage of lung tissue and pulmonary edema, and inhibiting inflammatory reaction.

However, several limitations of this study should be acknowledged. First, the observation period was relatively short (12 h), mainly reflecting the acute phase of oleic acid–induced lung injury. Longer observation and follow-up studies are needed to assess the sustained efficacy and safety of deslanoside. Although no cardiac adverse events were observed at the tested dose, its narrow therapeutic window warrants future evaluation with electrocardiographic monitoring and histopathological assessment to ensure safety. Second, pharmacokinetic data were not obtained, and the pharmacokinetic–pharmacodynamic (PK/PD) relationship remains undefined. Future studies incorporating LC–MS–based drug quantification in serum and lung tissue will help correlate exposure with efficacy and guide optimal dosing strategies. Given deslanoside’s limited therapeutic index, clinical translation must proceed cautiously. In patients with ALI/ARDS, especially those with cardiovascular comorbidities, careful dose titration, cardiac monitoring, and electrolyte management will be essential. Novel pulmonary delivery systems—such as inhaled or nanoparticle formulations—may further improve safety by minimizing systemic exposure. Third, no positive control (dexamethasone or digoxin) was included in this study. Comparative studies with established anti-inflammatory agents will help position deslanoside’s efficacy and clarify its mechanistic distinctions based on Na^+^/K^+^-ATPase–mediated signaling. Additionally, specific epithelial and endothelial injury markers were not analyzed. Future work will examine alveolar apoptosis (caspase-3, TUNEL) and tight junction proteins (occludin, claudin-5, ZO-1) to determine whether deslanoside directly protects the alveolar–capillary barrier. Likewise, anti-inflammatory mediators such as IL-10 and macrophage polarization markers (M1/M2) should be evaluated to elucidate whether deslanoside promotes inflammation resolution and tissue repair.Finally, mechanistic conclusions remain inferential. Further molecular studies, including Western blotting, RT-qPCR, and immunofluorescence, are required to validate the involvement of NF-κB and NLRP3 pathways and confirm the proposed mechanism of action.

## Conclusion

Deslanoside showed significant therapeutic effect on acute lung injury induced by oleic acid in rabbits, which could effectively reduce pulmonary edema and hemorrhage caused by acute lung injury, and effectively inhibit inflammatory reaction, thereby alleviating the degree of lung injury.

## Data Availability

The original contributions presented in the study are included in the article/supplementary material, further inquiries can be directed to the corresponding author.
